# Piccolo is essential for the maintenance of mouse retina but not cochlear hair cell function

**DOI:** 10.18632/aging.202861

**Published:** 2021-04-21

**Authors:** Peipei Li, Zhuchun Lin, Yachun An, Jing Lin, Aizhen Zhang, Shuangyan Wang, Hailong Tu, Jie Ran, Jinpeng Wang, Yu Liang, Ziyi Liu, Chao Ye, Xiaolong Fu, Jiangang Gao

**Affiliations:** 1 School of Life Science and Key Laboratory of the Ministry of Education for Experimental Teratology, Shandong University, Jinan 250100, China; 2 First People's Hospital of Jinan, Jinan 250011, China; 3 The Waksman Institute of Microbiology, Rutgers University, Piscataway, NJ 08854, USA; 4 College of Life Sciences, Shandong Normal University, Jinan 250014, China; 5 MOE Key Laboratory for Developmental Genes and Human Disease, Jiangsu Province High-Tech Key Laboratory for Bio-Medical Research, Institute of Life Sciences, Southeast University, Nanjing 210096, China

**Keywords:** piccolo, mouse, cochlea, CRISPR/Cas9, retina

## Abstract

Piccolo is a presynaptic protein with high conservation among different species, and the expression of Piccolo is extensive in vertebrates. Recently, a small fragment of Piccolo (Piccolino), arising due to the incomplete splicing of intron 5/6, was found to be present in the synapses of retinas and cochleae. However, the comprehensive function of Piccolo in the retina and cochlea remains unclear. In this study, we generated *Piccolo* knockout mice using CRISPR-Cas9 technology to explore the function of Piccolo. Unexpectedly, whereas no abnormalities were found in the cochlear hair cells of the mutant mice, significant differences were found in the retinas, in which two layers (the outer nuclear layer and the outer plexiform layer) were absent. Additionally, the amplitudes of electroretinograms were significantly reduced and pigmentation was observed in the fundoscopy of the mutant mouse retinas. The expression levels of Bassoon, a homolog of Piccolo, as well as synapse-associated proteins CtBP1, CtBP2, Kif3A, and Rim1 were down-regulated. The numbers of ribbon synapses in the retinas of the mutant mice were also reduced. Altogether, the phenotype of *Piccolo*-/- mice resembled the symptoms of retinitis pigmentosa (RP) in humans, suggesting *Piccolo* might be a candidate gene of RP and indicates *Piccolo* knockout mice are a good model for elucidating the molecular mechanisms of RP.

## INTRODUCTION

Coding of auditory and visual information occurs at synapses that are driven by receptor potentials of hair cells and photoreceptors, respectively [1]. Synaptic vesicles dock and fuse at a specific region of the presynaptic plasmalemma defined by a unique and tightly regulated complex meshwork of proteins, i.e., the cytomatrix at the active zones (CAZ) [2–5]. CAZ is composed of an evolutionarily conserved protein complex [6]. Piccolo is one of the CAZ proteins that participate in the functional connection between the dynamic F-actin cytoskeleton and synaptic vesicle recycling and therefore, may be involved in the release of neurotransmitters [7–10]. The *Piccolo* gene is located on chromosome 7 in mice, contains 25 exons, and encodes 5068 amino acids [11]. The protein has a molecular weight of ~550 kDa and contains one glutamine-rich Q domain, two zinc finger domains, three coiled-coil domains, a PDZ domain, and two C2 domains (C2A and C2B) [11]. There are three splicing variants of *Piccolo* that encode amino acids, and all three share the same start codon [11, 12]. One of the splicing variants has a stop codon in exon 20 and encodes a 530 kDa protein that does not include the C2B domain. Another splicing variant encodes a 550 kDa protein that has a stop codon in exon 25. The third splicing variant was recently discovered in the retina and cochlea and occurs due to the non-splicing of intron 5/6, which is involved in the function of ribbon synapses [12]. This third version of *Piccolo* was named *Piccolino* and encodes a ~350 kDa protein [12]. *In situ* proximity junction measurements show Piccolino has lost the site present in Piccolo that interacts with Bassoon, Munc13, ELKS/CAST, RIM and L-type Ca2+ channels, suggesting that the function of Piccolino in ribbon synapses differs from that of Piccolo in conventional synapses [12, 13].

Ribbon synapses are found in cochlear hair cells, vestibular hair cells and photoreceptors in mammals [14, 15]. In the inner ear, the shape, number, and size of ribbon synapses, as well as their connectivity with afferent neurons vary greatly in the hair cells of various organs and often within the same organ [16–18] and even within the same hair cell [19–22]. In response to a graded receptor potential, the hair cells of the auditory and vestibular epithelia of the inner ear release glutamate. One nerve terminal links one ribbon in an inner hair cell (IHC); therefore, every CAZ enables one spiral ganglion neuron to accept the sole information from an IHC synapse allowing acoustic information to transmit rapidly without rundown through multiple ribbon synapses in IHC [23]. The vertebrate retina is a layered structure, this feature is called retinal lamination and disorganization of retinal lamination often leads to impaired overall organ function. The development of the vertebrate retina is accompanied by the formation of a synaptic connection between the nerve layers [24]. The nerve layer of the retina is composed primarily of six major types of differentiated neurous: retinal ganglion cells, the cone and rod photoreceptors, bipolar cells, amacrine, and horizontal cells [25, 26]. The outer nuclear layer(ONL) consists mainly of photoreceptor cells that use glutamate as a neurotransmitter and synapse onto second order glutamatergic bipolar cells at the outer plexiform layer (OPL) [27]. Bipolar cells can be divided into rod bipolar cells and cone bipolar cells and cone bipolar cells contact retinal ganglion cells and amacrine cells at the inner plexiform layer (IPL). The ganglion cells are output neurons of the retina [27, 28]. Mutations within any of the molecules responsible for these visual processes could cause several types of retinal pigment epithelium degenerative diseases [29–33].

Mukherjee et al. found that *Piccolo* knockout mice exhibited increased postnatal mortality, and obvious reduction in synaptic vesicle clustering in double *bassoon*/*piccolo* deficient synapses [34]. In recent years, Regus-Leidig et al. discovered Piccolino, a splice variant and small segment of Piccolo arising due to the incomplete splicing of intron 5/6 specifically expressed in the sensory ribbon synapses of the eye and ear. Thus, the previous *Piccolo* knockout mice did not abolish the function of Piccolino. Furthermore, using adeno-associated vector-mediated RNA interference technology, it was observed that the downregulation of Piccolino caused ultrastructural changes in mouse retinal photoreceptor cells and changed the morphology of the ribbon synapses from plate-shaped to membrane-bound spheres [13]. Moreover, the knockdown of *Piccolo* resulted in a decrease in *Piccolo* mRNA to 58% of the wildtype control value and an inability of ribbon synapses to dynamically assemble and disassemble in transfected cells [13]. Despite this information, a complete *Piccolo* knockout animal model has not been reported, and the specific mechanism of Piccolo in retinal ribbon synapses is still unclear.

In this study, we used CRISPR-Cas9 technology to fully delete *Piccolo* to obtain a more effective knockout model and elucidate important biological functions and molecular mechanisms of Piccolo in the retina. We found that Piccolo is essential for the functional maintenance of the mouse retina but not IHC of the inner ear. The *Piccolo* knockout mice also showed defective electroretinograms (ERGs) and abnormal retinal anatomy. The phenotype of *Piccolo* knockout mice resembled the symptoms of retinitis pigmentosa (RP) in humans, suggesting that *Piccolo* might be a candidate gene of RP.

## RESULTS

### Generation of *Piccolo* knockout mouse using CRISPR/Cas9 technology

**Figure 1:**
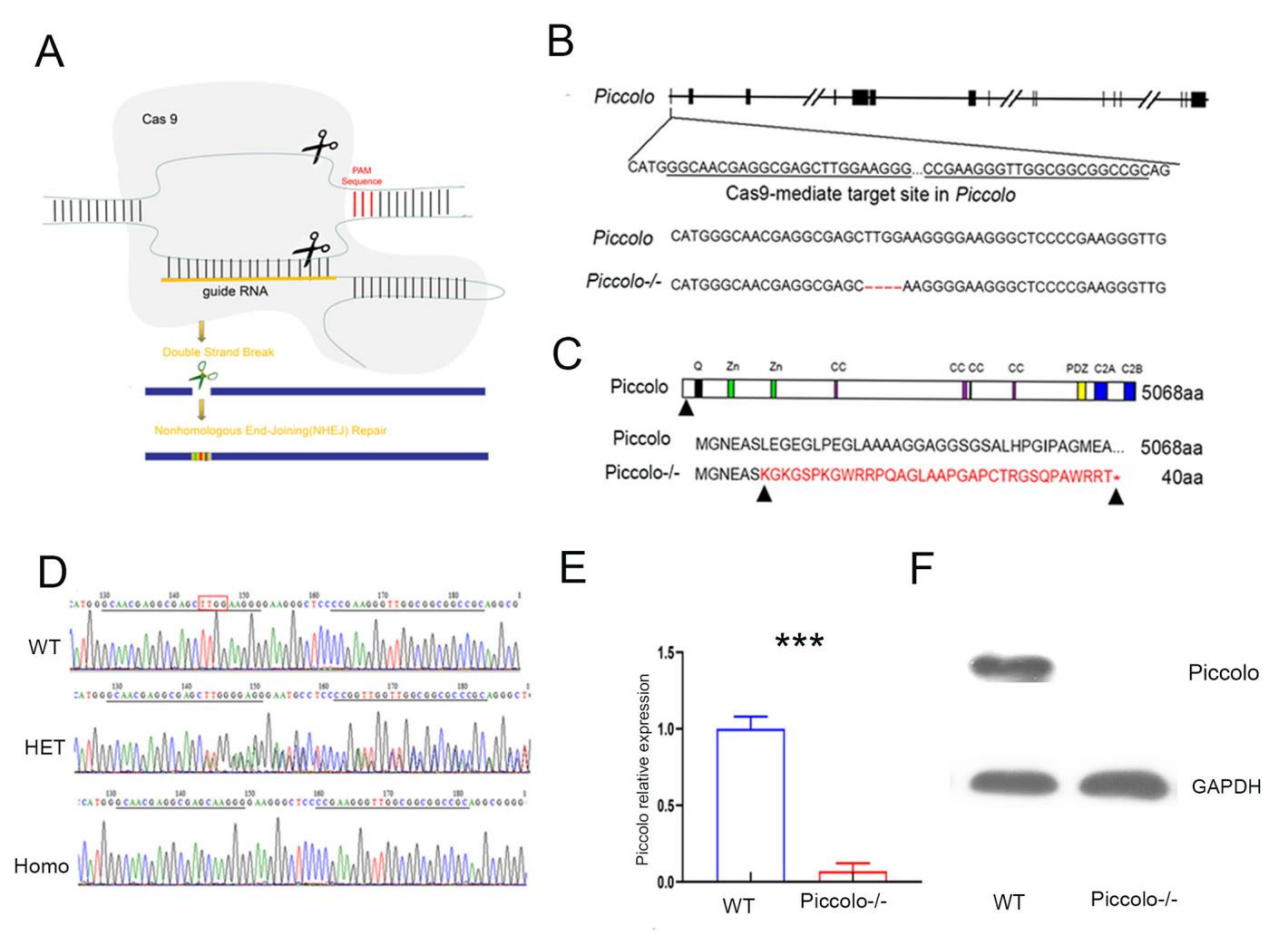
**Generation of *Piccolo*-/- mouse.** (**A**) Schematic diagram of the CRISPR/Cas9 knockout strategy. (**B**) Schematic illustration of Cas9-mediated targeting of the mouse *Piccolo* gene (sgRNA and PAM sequences are underlined) and comparison of *Piccolo* DNA sequences in wild type and *Piccolo*-/- (4 bp deletion) mice. (**C**) Schematic illustration of the frameshift mutation in *Piccolo*-/- mice. The altered amino acid sequence is marked in red. “*” indicates a premature stop codon. The translation of protein is terminated at the point of the arrow. Also shown are the Piccolo wild type protein sequence and domain organization. (**D**) Sequencing chromatograms of *Piccolo* in wild type (WT) mice, heterozygous (HET; *Piccolo+/*-) mice, and homozygous (Homo; *Piccolo*-/-) mice. The red box indicates the bases missing in the homozygous (Homo) *Piccolo*-/- mice. (**E**) Relative *Piccolo* expression in *Piccolo*-/- and WT mice as measured by Q-PCR. (**F**) Western blot analysis of Piccolo protein in the retina of *Piccolo*-/- and WT mice. GADPH was used as the loading control.

To investigate the function of Piccolo in the retina and cochlea, we created a full *Piccolo* knockout mouse model using CRISPR/Cas9 technology (Figure 1A). We designed two targets on exon 1 of *Piccolo* (Figure 1B) then ligated sgRNA containing the target sequence into the PX330 plasmid containing the Cas9 gene to obtain the Piccolo-PX330-sgRNA plasmid. A fertilized egg in the pronuclear stage was injected with the constructed plasmid and transplanted into a pseudopregnant CD1 mouse. The offspring obtained were founder mice (F0) mice. PCR analysis revealed various mutation types such as deletions of 28 bp, 31 bp, 4 bp, and 16 bp in the F0 generation. The genotypes of the homozygous mice were confirmed by PCR sequencing (Figure 1C). We used mutant mice with the 4 bp deletion in later experiments, and the number of amino acids reduced from 5048 to 40. Since the targets are on exon 1, all splices (*Piccolo* and *Piccolino*) were knocked out simultaneously. We used Q-PCR to determine whether the *Piccolo* gene was knocked out at the mRNA level and found that the *Piccolo* mRNA was dramatically decreased in the retinal nerve layer of *Piccolo*-/- mice compared with the wild- type controls (Figure 1E). Additionally, western blot results confirmed the *Piccolo* knockout mice at the protein level (Figure 1F). Taken collectively, these results suggest that these *Piccolo-/-* mice are an ideal model to study Piccolo function.

### The auditory function of *Piccolo-/-* mice was not affected, while their visual function was seriously impaired

**Figure 2:**
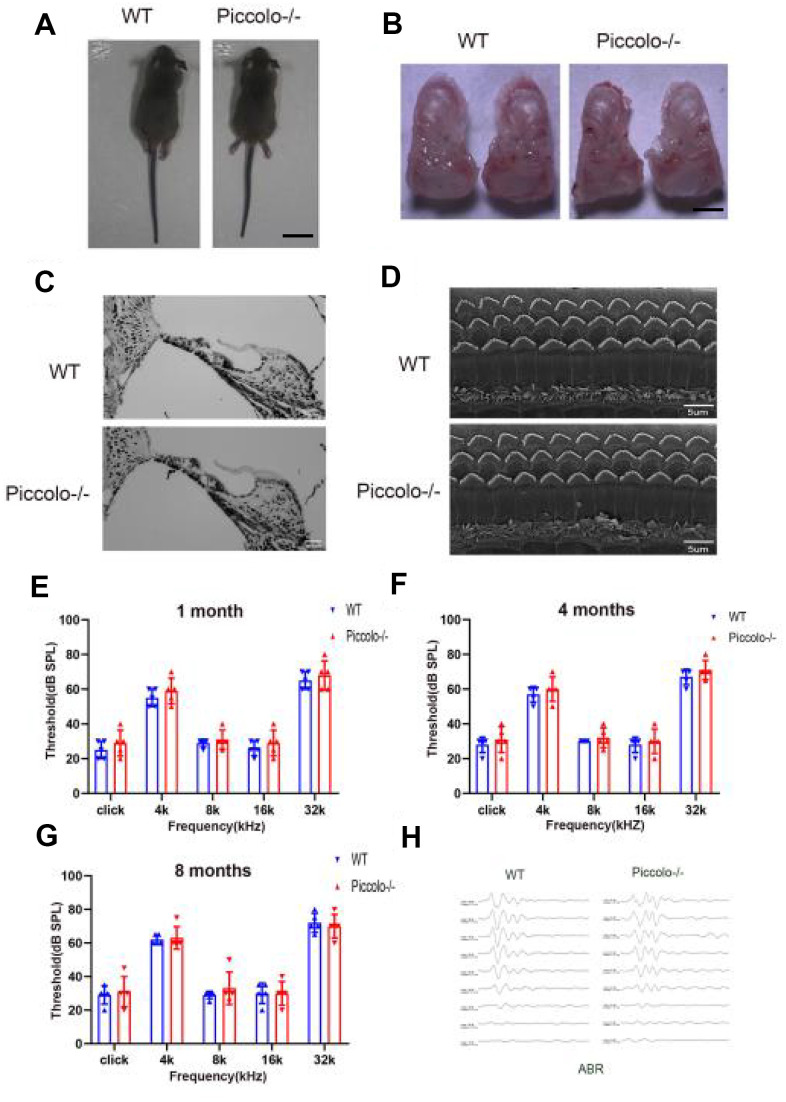
**Auditory function of *Piccolo*-/- mice.** (**A**) Gross morphology of *Piccolo*-/- and wild type (WT) mice at the age of one month (P30). Scale bar = 2cm. (**B**) Overall appearance of cochleae from *Piccolo*-/- and WT mice at P30. Scale bar = 15mm. (**C**) H&E staining of cochlear sections in *Piccolo*-/- and WT mice at P30. Scale bar = 20 μm. (**D**) Scanning electron microscopic images of cochlear hair bundles in *Piccolo*-/- and WT mice at P30. Scale bar = 5 μm. (**E**–**G**) ABR thresholds of *Piccolo*-/- (n = 3) and WT (n = 3) mice (at the age of one month, 4 months and 8 months, respectively) to click stimuli and to 4, 8, 16, and 32 kHz stimuli. Error bars = Means ± SD. (**H**) Representative ABR recording from control (n = 5) and *Piccolo*-/- (n = 3) mice at P30. The asterisk indicates a significant difference between WT and *Piccolo*-/- mice. *P < 0.05, **P < 0.01, by two-tailed Student’s t test. Error bars = Mean ± SD.

**Figure 3:**
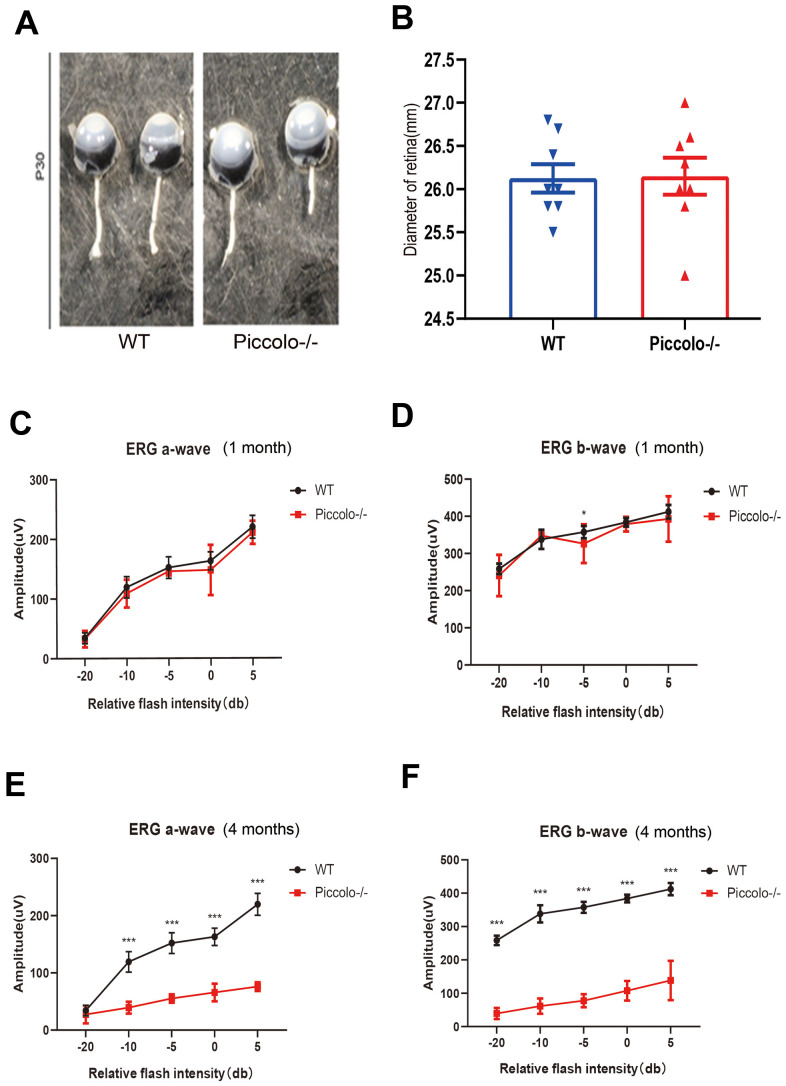
**Structure and function of retina in wild type and *Piccolo*-/- mouse.** (**A**) Eyes from wild type (WT) and *Piccolo*-/- mice. (**B**) Mean diameters of eyeballs from WT (n = 8) and *Piccolo*-/- (n = 8) mice. (**C**) ERG recording a-wave amplitudes from WT (n=6) and *Piccolo*-/- (n=6) mice at P30. (**D**) ERG recording b-wave amplitudes from WT (n=6) and *Piccolo*-/- (n=6) mice at P30. (**E**) ERG recording a-wave amplitudes from WT (n=6) and *Piccolo*-/- (n=6) mice at 4 months. (**F**) ERG recording b-wave amplitudes from WT (n=6) and *Piccolo*-/- (n=6) mice at 4 months. The asterisk indicates a significant difference between WT and *Piccolo*-/- mice. *P < 0.05, **P < 0.01, by two-tailed Student’s t test. Error bars = Mean ± SD.

The *Piccolo*-/- mice generated for this study were viable and fertile and appeared normal compared to the littermate controls throughout their lifespan (Figure 2A), which is in stark contrast to the *Piccolo*-/- mice reported in a previous study [34]. Due to the fact that Piccolino is specifically expressed in the eye and cochlea, we analyzed their structure and function of wild-type and *Piccolo*-/- mice, respectively. The overall appearance of the cochleae showed no abnormalities in the *Piccolo*-/- mice at P30 (30 days after birth) (Figure 2B). No significant differences in the hair cell expression pattern was found in *Piccolo*-/- mice compared with the wild- type controls (Figure 2C, 2D). In addition, ABR results showed no significant difference between *Piccolo*-/- mice and wild- type mice when checked at 1, 4, and 8 months of age, respectively (Figure 2E–2H). The peak I amplitudes of the ABR waves and the number of ribbon synapse also show no significant difference between two groups (Supplementary Figure 1A, 1B). These results indicate that the hearing of *Piccolo*-/- mice is normal. We also checked the eyes between the two groups and found no significant difference in the appearance and size of the eyes in *Piccolo*-/- mice compared with the control mice at one month of age (Figure 3A, 3B). ERG analysis was performed to measure retinal function. ERG analysis was performed to measure retinal function in *Piccolo*-/- mice and wild-type mice at 1, 4, and 8 months of age, respectively. The amplitudes of both the a-wave and b-wave in *Piccolo*-/- mice were actually the same compared to wild-type controls under different flash intensity stimuli at P30, however, they decreased significantly when checked at 4 months of age, indicating that phototransduction is disturbed in *Piccolo*-/- mice at 4 month of age (Figure 3C–3F).

### The retinal anatomy is abnormal in *Piccolo*-/- mice

**Figure 4:**
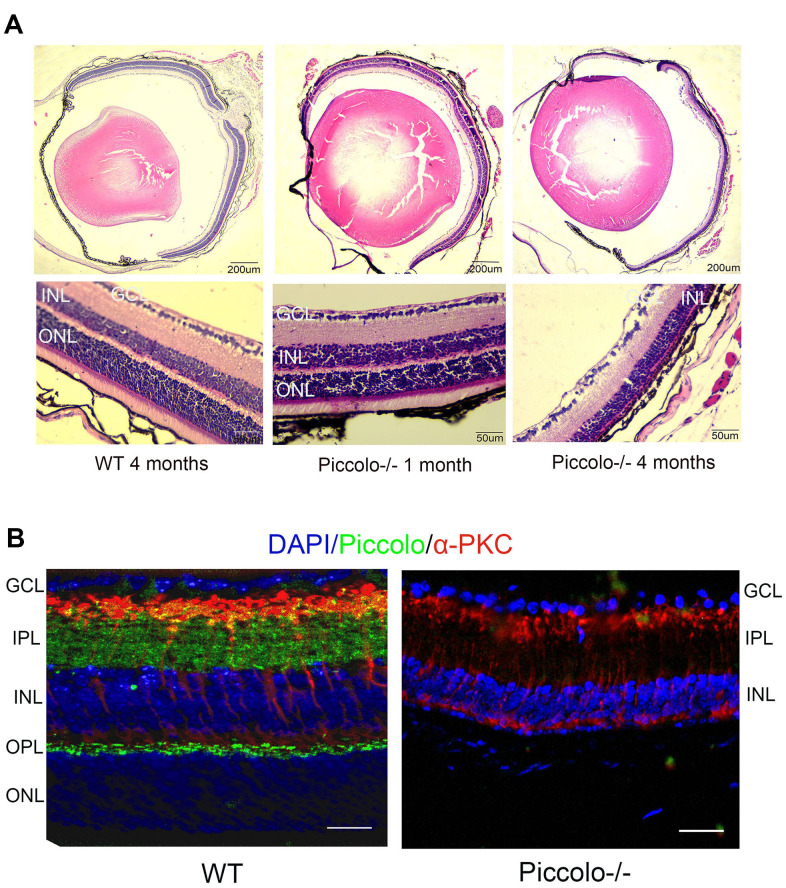
**Retinal anatomy in *Piccolo*-/- mice.** (**A**) H&E stained paraffin sections of retinas from *Piccolo*-/- and wild type (WT) mice. The image below represents the magnification of the upper image. (**B**) Immunofluorescence staining with DAPI, α-PKC (marker for bipolar cells), and anti-Piccolo antibodies in the retina of *Piccolo*-/- and WT mice. Scale bars: 20 μm.

**Figure 5:**
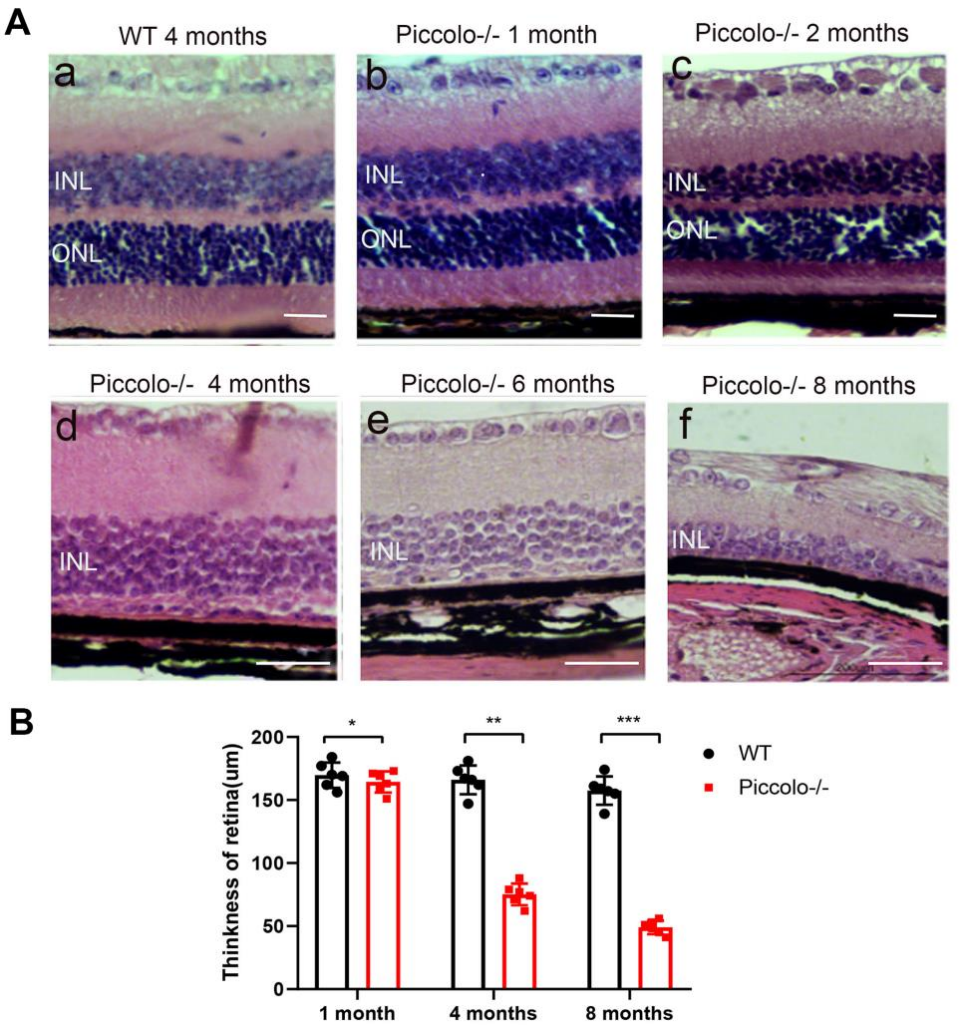
**Retinal anatomy in wild type and *Piccolo*-/- mice.** (**A**) H&E stained sections of retinas from *Piccolo*-/- and wild type mice at one, two, four, six and eight months of age, respectively. (**B**) The thickness of retina in *Piccolo*-/- and wild type (WT) mice (n = 6 per group) at 1, 4 and 8 months; ***P < 0.001, by two-tailed Student’s t test.

**Figure 6:**
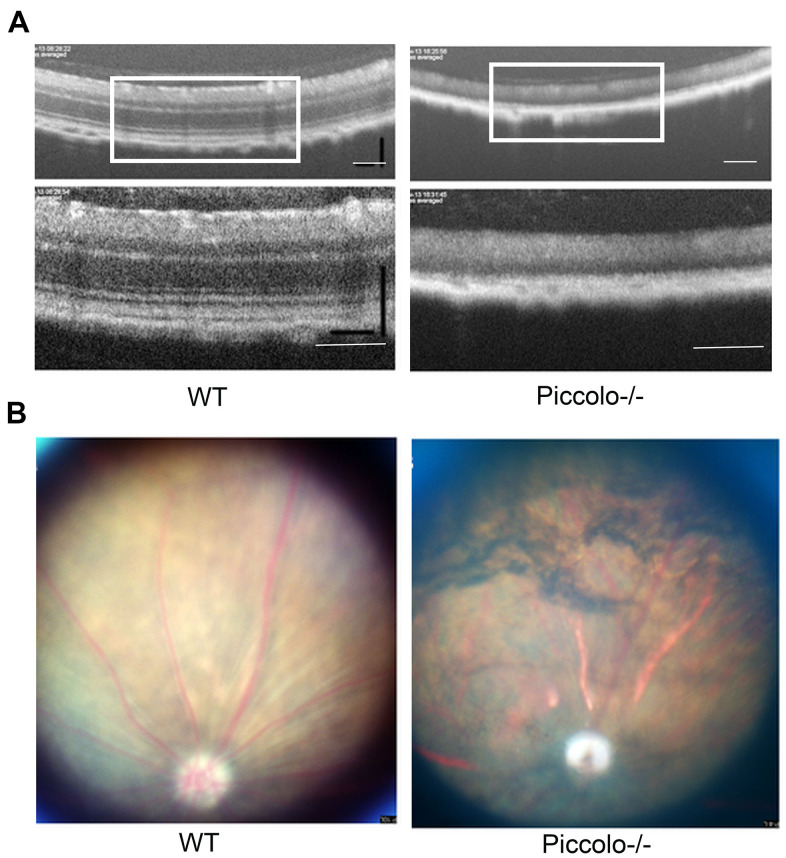
**Examination of the retinal fundus in *Piccolo*-/- mice.** (**A**) Optical coherence tomography (OCT) of the retina in *Piccolo*-/- mice compared with the wild type (WT) mice at P30; Scale bars: 100 μm. (**B**) Retinal fundus imaging in one-month-old WT and *Piccolo*-/- mice.

Due to the fact that the function of the retina was affected in Piccolo-/- mice, we hypothesized that the retinal anatomy was affected as well. Therefore, paraffin sections of eyes from in the *Piccolo*-/- and wild-type mice at the age of 1 and 4 months were stained with H&E. We observed the retina of the *Piccolo-/-* mice at P30 was comparable with the wild-type controls. However, the retina of the *Piccolo-/-* mice become thinner at 4 months (Figure 4A). To determine which layer was lost in the retinas of *Piccolo*-/- mice, we performed immunofluorescence staining of α-PKC, a marker for bipolar cells which lie in inner nuclear layer of retina. Thus, the results showed that the inner nuclear layer of retina was intact, while the outer nuclear layer was lost in *Piccolo*-/- mice (Figure 4B). Further study showed that the anatomy of retina in *Piccolo*-/- mice degenerated with age (Figure 5A, 5B). Collectively, these results suggest that the degeneration of photoreceptors is gradual in *Piccolo*-/- mice, resembling the symptoms of RP in humans. Optical coherence tomography (OCT) is an optical diagnostic technique, which uses the interference of light to observe biological tissue structure. OCT, performed on *Piccolo*-/- and wild-type mice, confirmed retinas were thinner in *Piccolo*-/- mice compared with wild-type mice at 4 months(Figure 6A) and *Piccolo*-/- mice at 4 months were found to have pigmentation in the fundus, a characteristic of RP which was not observed wild-type mice (Figure 6B).

### The morphology, location, and number of ribbon synapses were altered in *Piccolo*-/- mice

**Figure 7:**
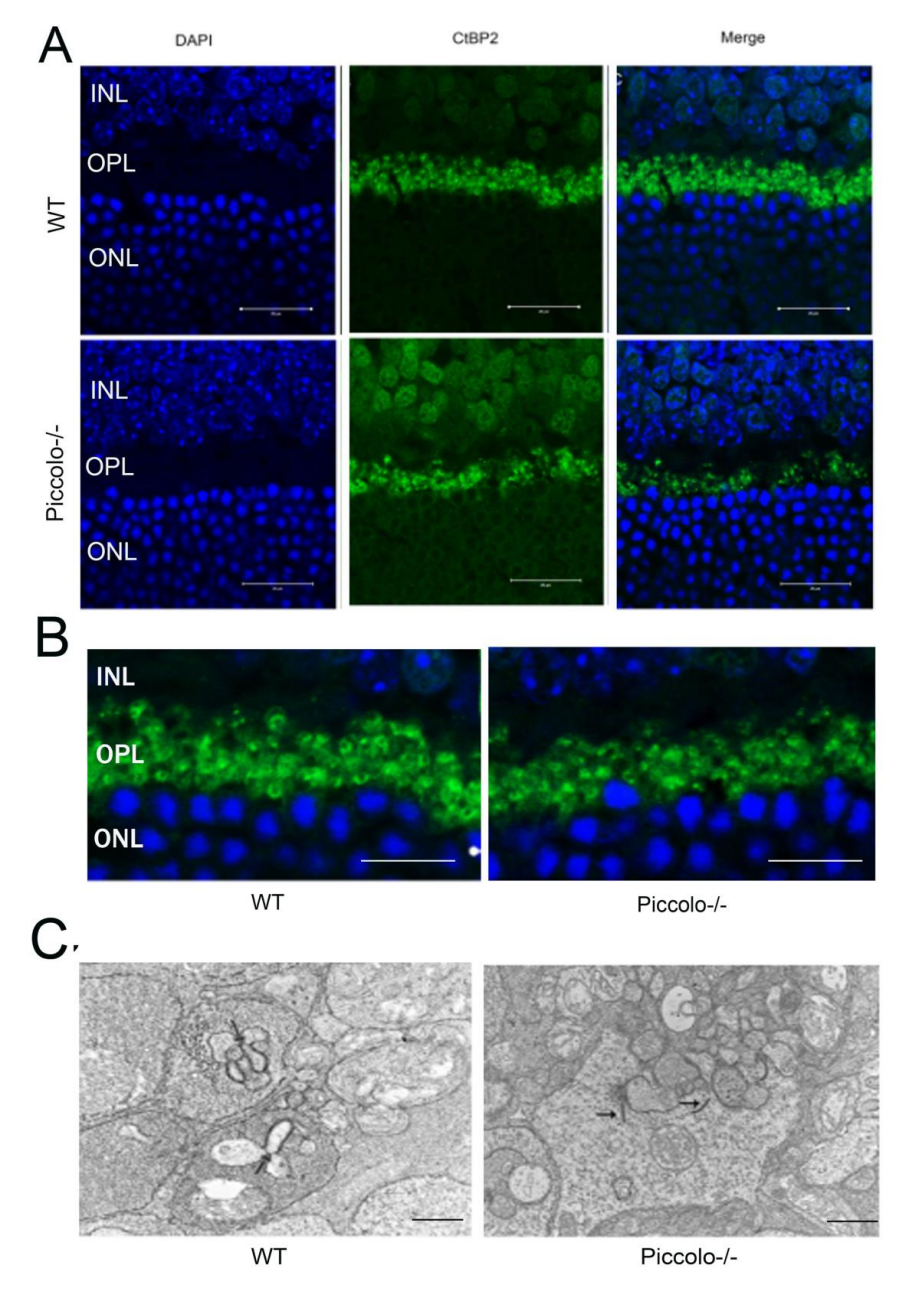
**The morphology, location, and numbers of ribbon synapses in *Piccolo*-/- mice.** (**A**) Immunostaining with anti-CtBP2 antibodies of retina cryosections from wild type (WT) and *Piccolo*-/- mice at P30. DAPI stains nuclei. Scale bars: 20 μm. (**B**) The morphology of ribbon synapses in the *Piccolo*-/- and WT mice. Scale bars: 10 μm. (**C**) The cytoplasmic location of retinal ribbon synapses (indicated by arrows) in *Piccolo*-/- mice. Scale bars: 1 μm.

It has been reported that when *Piccolino* is downregulated *in vivo* via an adeno-associated virus-based RNA interference approach, the ribbon synapses have striking changes in morphological in ultrastructure [13]. In view of this, we hypothesized that the knockout of *Piccolo* would also affect the morphology of ribbon synapses. Therefore, cryosections of retinas from P30 *Piccolo*-/- mice and wild-type mice were immunostained with antibodies against CtBP2 (CtBP2 is a marker for ribbon synapses). In photoreceptor terminals in retinal areas from wild type mice, CtBP2 staining showed the typical horseshoe shape associated with ribbon structure. In contrast, CtBP2 staining in the retinal areas from *Piccolo*-/- mice revealed very few horseshoe-shaped structures, and staining appeared mostly as small fluorescent puncta (Figure 7A, 7B). We also found that the number of ribbon synapses in *Piccolo*-/- mice greatly decreased (Figure 7A). Using transmission electron microscopy to analyze the retinas of one-month old mice major changes in the location of ribbon synapses of retina in the *Piccolo*-/- mice were observed. Thus, the ribbon synapses in the *Piccolo*-/- mice were not anchored to the presynaptic active zones, but floated freely in the cytoplasm (Figure 7C).

### The expression of ribbon-associated proteins was decreased in *Piccolo*-/- mice

**Figure 8:**
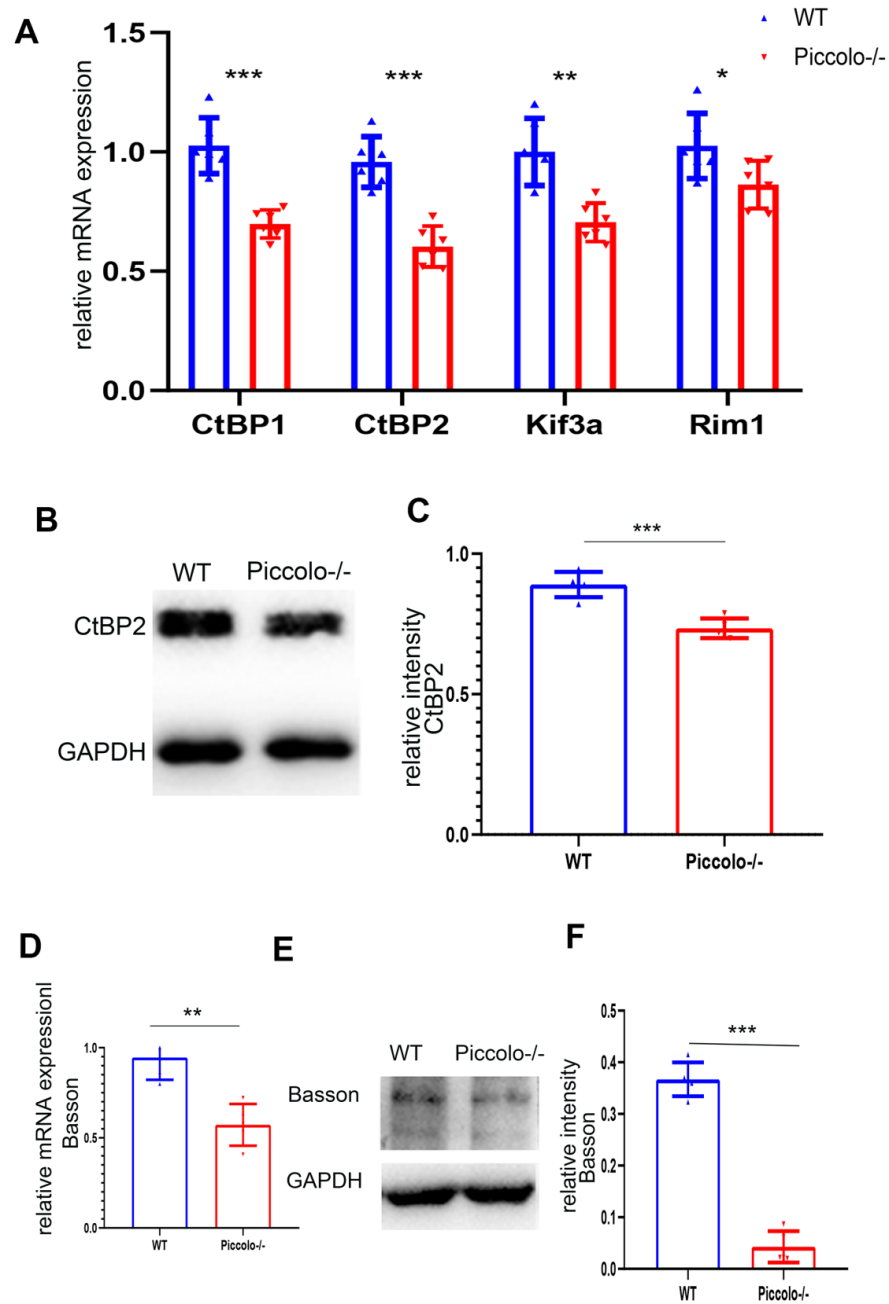
**Expression levels of synaptic ribbon-related proteins in *Piccolo*-/- mice.** (**A**) Relative mRNA levels of ribbon-related genes in *Piccolo*-/- and wild type (WT) mice. n=5. (**B**) Western blot analysis of CtBP2 in *Piccolo*-/- and WT mice (GADPH was used as the loading control.) n=5. (**C**) Relative CtBP2 band intensities normalized to GADPH. n=5. (**D**) Relative mRNA levels of *Bassoon* in *Piccolo*-/- and WT mice. n=5. (**E**) Western blot analysis of Bassoon in *Piccolo*-/- and WT mice. (GADPH was used as the loading control.) n=5. (**F**) Relative Bassoon band intensities normalized to GADPH. Data represent the means ± SD. **P < 0.01, ***P < 0.001, by two-tailed Student’s t test, n=5.

Since the morphology and location of most ribbon synapses in the retina were altered in the *Piccolo*-/- mice, we suspected the expression level of ribbon-associated proteins was also affected at P30. Therefore, we examined the expression levels of ribbon-associated genes *RIBEYE/CtBP2*, *CtBP1/BARS*, *KIF3A* and *RIM1* using Q-PCR. The expression levels of *CtBP1, CtBP2, KIF3A* and *RIM1* in the *Piccolo*-/- mice were significantly decreased (Figure 8A). In view of the fact that *RIM1*, *CtBP2* and *Kif3a* mutations are associated with RP [35–40], further indicating that *Piccolo* is a candidate gene of RP.

### *Bassoon* expression is decreased in *Piccolo*-/- mice

*Bassoon* and *Piccolo* encode highly homologous proteins. The absence of Bassoon affects the localization of ribbon synapses, making them unable to anchor at the presynaptic active zones; this resembles the phenotype of ribbon synapses in *Piccolo*-/- mice [41]. Therefore, the mRNA and protein levels of Bassoon in the *Piccolo*-/- mice were assessed using quantitative real-time PCR (Q-PCR) and western blot at P30, respectively. The mRNA expression level of *Bassoon* was significantly decreased (Figure 8B), and the protein level of Bassoon was also decreased (Figure 8C).

## DISCUSSION

### We obtained a more effective *Piccolo* knockout animal model

Previous studies of the *Piccolo* gene have focused on its role in the central nervous system, such as the brain and cerebellum [42–44], and little is known about the specific function of Piccolo in the retina and cochlea. To study the function of Piccolino in the ribbon synapses of the retina and cochlea, we fully deleted the *Piccolo* gene in mice using the CRISPR-Cas9 technique and obtained a more effective knockout model than previously reported [13, 34, 45]. This technique deleted *Piccolo* and *Piccolino* simultaneously. The previously reported *Piccolo* knockout mice all contained the Q domain, a region that engages with clathrin-mediated endocytosis [8, 11]. Additionally, this region interacts with the actin binding protein mAbp1 and serves to localize Abp1 to the CAZ where it can participate in creating a functional connection between the dynamic actin cytoskeleton and synaptic vesicle recycling. This suggests that Piccolo may also be indirectly involved in synaptic vesicle endocytosis [46, 47]. This is an important reason why the phenotype of our *Piccolo* knockout mice differs from that of other reported *Piccolo* knockout mice. Another reason may be that our knockout mice have different strain backgrounds from other knockout mice.

### Phenotypic differences in cochleae and retina of *Piccolo* knockout mice

In the present study, Piccolo presented in the two synaptic layers of the retina, the outer plexiform layer and the inner plexiform layer. Piccolo also showed strong staining in the ribbon synapses of cochlear IHCs [12]. Nevertheless, the knockout of *Piccolo* did not cause hearing problems; however, it did lead to serious visual problems. We show that the phenotypic differences between cochlea and retinal function are likely reflective of differences in the composition of presynaptic proteins. In other words, the architecture of synapses varies greatly, and the structural differences correlate with the specific function of different genes [1, 14, 15, 48, 49]. Another possible explanation for this may be the presence of a redundant protein compensating for Piccolo loss in the cochlear IHCs. In this study, we found that Piccolo is present in both conventional chemical synapses and ribbon synapses in the retina. It is noteworthy that the conventional chemical synapses in retina did not show obvious morphological differences in *Piccolo* knockout mice compared with wild type mice. In stark contrast, most ribbon synapses in the outer plexiform layer of retinas in *Piccolo* knockout mice displayed spherical structures not observed in the wild type mice. The difference in the molecular composition, including *Piccolo* and affected downstream genes, may account for the phenotype displayed by the *Piccolo* knockout mice.

### Changes in expression of bassoon and other synapse-related proteins

Piccolo and Bassoon, two structurally related presynaptic cytomatrix proteins, showed strong punctate immunofluorescence in the outer and the inner plexiform layers of the retina. In our study, Bassoon was down-regulated at the mRNA and protein levels in *Piccolo*-/- mice. In another study, the expression of Piccolo is also decreased in *Bassoon* knockout mice [50], suggesting that the relationship between the two proteins is not simply upstream or downstream expression or function. However, there may be a synergistic effect between Bassoon and Piccolo in the maintenance of ribbon synapse function in the CAZ [9, 51–53]. Studies have reported that at photoreceptor ribbon synapses, Piccolo and Bassoon epitopes are spatially segregated along the whole extent of the synaptic ribbon [54, 55]. Bassoon and Piccolo were shown to co-localize in retinal ribbon synapses; however, Bassoon is located at the bottom of the ribbon synapses, while Piccolo is located around the periphery of ribbon synapses [54, 55]. The difference in localization may explain the non-redundant functions of Piccolo and Bassoon in retinal ribbon synapses and suggest that the interrelationship between Piccolo and Bassoon might be via interaction with other synapse-associated proteins.

### *Piccolo* knockout mouse can be used as an ideal model of retinitis pigmentosa

RP is a hereditary eye disease characterized by the progressive loss of retinal photosensitive cell and pigment epithelial cell function [29, 56]. RP is a common blinding eye disease worldwide, for which there is no effective cure. Thus, it is necessary to establish a mouse model of RP to clarify the specific molecular mechanism(s) of RP; the homology between mouse and human genomes is very high. Although gene function is not exactly the same between mouse and human, animal models can simulate human diseases and provide theoretical support for understanding the pathogenesis and developing treatment of this disease.

In this study, our *Piccolo*-/- mouse model showed progressive degeneration of photoreceptor cells, pigmentation in the fundus, and down-regulated expression of ribbon synapse related proteins. It has been reported that *Kif3a* knockout mice and *Kif3a* knockout zebrafish also have the RP phenotype, i.e., retinal thinning and loss of the outer nuclear layer [39, 57]. The mutation of Rim1 is also associated with RP [42, 43]. The phenotype produced in our knockout mice was also similar to RP.

In summary, we used CRISPR-Cas9 technology to obtain a more efficient *Piccolo* knockout mouse model than previously available. Retinal thinning and retinal pigmentation were observed in our *Piccolo*-/- mice. ERGs showed decreased visual function in *Piccolo*-/- mice. These phenomena indicated that *Piccolo*-/- mice had RP in morphology and function; however, the specific mechanism underlying the phenotype needed further study. Changes in ribbon morphology and related protein expression and localization suggested that the ribbon synapses were abnormal, and this lead to progressive degeneration of photoreceptor cells. Although several papers have reported that CAZ protein mutation cause photoreceptor cell death, the molecular mechanism of photoreceptor cell death in *Piccolo* knockout mice remains unknown. The *Piccolo*-/- mice showed an RP phenotype, indicating this gene may be a potential RP candidate gene. In conclusion, the *Piccolo*-/- mice can serve as a good animal model of RP and potentially provide theoretical support for the treatment of RP.

## MATERIALS AND METHODS

### Experimental animals

We used CRISPR-Cas9 genome-editing technology to obtain the *Piccolo* knockout mouse model in the CBA/CaJ mouse line. The CRISPR-Cas9 genome-editing technology was used as previously described [58, 59]. The pX330 plasmid was obtained from Addgene (Plasmid ID: #42230). We designed a pair of oligonucleotides containing the target sequences (GCAACGAGGCGAGCTTGGAA and AAGGGTTGGCGGCGGCCGC). The oligonucleotides were annealed and ligated into BbsI-digested pX330. The pX330 plasmid containing the sgRNA and Cas9 mRNA was purified and eluted in RNase-free water. The CBA/CaJ female mice were superovulated and mated with CBA/CaJ male mice. The fertilized eggs were removed from oviducts the following day. The pX330 plasmid with inserts (5 ng/μL) was microinjected into the pronuclei of the fertilized eggs, and eggs were incubated at 37° C for 10 minutes. The eggs were then transferred into the oviducts of pseudopregnant CD-1 female mice. The mice born nineteen days later were referred to as F0 knockout mice. We searched for the off-target locus using the web-based analysis tool Off-Spotter on the CRISPR design site (http://crispr.mit.edu) and found no off-target changes occurred in the *Piccolo*-/- mice. The *Piccolo-/-* mice and WT mice were fed in SPF animal house of College of Life Sciences, Shandong University. The temperature is controlled at 22-24° C and mice were kept on a 12-hour light: 12-hour dark cycle, with food and water available ad libitum.

### Auditory brainstem response (ABR)

ABR was performed in a soundproof room to determine the hearing thresholds of the mice. Mice were deeply anesthetized with 0.007 g/mL pentobarbital sodium by intraperitoneal injection (50 mg/kg body weight). Three electrodes were inserted subcutaneously in the anesthetized mice through the cranial vertex, underneath the ear, and posteriorly near the tail, respectively. Hearing thresholds were measured using a Tucker-Davis Technologies System (TDT, USA) workstation running the *SigGen32* software (TDT, USA). Mice were subjected to click and tone burst stimuli at frequencies of 4, 8, 16, and 32 kHz. Auditory thresholds were defined by decreasing the sound intensity in 10 dB steps from 90 dB to 10 dB. The ABR threshold was defined as the lowest intensity at which reproducible waves were elicited clearly. *Piccolo*-/- mice were compared with their wild type littermates, and more than four animals per group were used in each experiment.

### Scanning electron microscopy

Cochleae from *Piccolo*-/- mice and wild type mice were dissected and fixed with 2.5% glutaraldehyde in phosphate buffered saline (PBS) at 4° C overnight and decalcified in 10% EDTA for 12 h. The sensory epithelium of the cochlear duct was then dissected and post-fixed in 1% osmium tetroxide for 2 h before dehydration in graded ethanol washes followed by critical point drying in an Autosamdri-815A (Tousimis). Samples were mounted on metal stubs, sputter coated with gold, and imaged using a JEOL 7000 field emission gun scanning electron microscope.

### Retinal protein extraction and western blot analysis

Wild- type and *Piccolo*-/- mice were sacrificed by cervical dislocation and their retinas (five mice for each group) promptly removed. Retinas were incubated in cell lysis buffer (10 mM Tris, pH = 7.4, 1% Triton X-100, 150 mM NaCl, 1 mM EDTA, 0.2 mM PMSF), and proteins were extracted using a homogenizer. Western blots were performed as described previously [60].

### RNA isolation and quantitative real-time PCR

Whole retinas were quickly isolated from one-month-old *Piccolo*-/- and wild- type littermate mice, and RNA was extracted using TRIzol Reagent (Invitrogen) according to the manufacturer’s protocol. Total RNA concentration and quality were assessed using an Eppendorf BioPhotometer plus. An A260/280 ratio of 1.9–2.1 and presence of two sharp peaks corresponding to the 18S and 28S RNA of all samples were indicators of high quality. cDNA was synthesized using the PrimeScript RT reagent Kit (cat. no. RR047A, TaKaRa, Japan). The quantitative real-time PCR (Q-PCR) was performed with Power SYBR Green PCR Master Mix (Takara) in a Bio-Rad thermal cycling system according to the manufacturer’s instructions. The amplification reaction mixture (10 μl) contained 0.5 μl of each primer in the SYBR Green system. (Primer sequences are listed in Table 1.) The target mRNA expression levels in wild type and *Piccolo*-/- mice were normalized to the housekeeping gene β-actin and then analyzed using the comparative CT method (also known as the ΔΔCT).

**Table 1:** Primer sequences of Q-pcr.

**Gene**	**Forward sequence**	**Reverse sequence**
Piccolo	AAAGCAAAACCAGTGTCGCC	TTGCGTCTTTTACTTGGTTGAAT
CtBP1	CTGGGGATCTAGGCATCGC	GTTCGTCGGTACAGGTTCAGG
CtBP2	TCGGTAGTGGCTACGACAAC	CGCCGATACAGATTGAGAATGT
Kif3a	ATCCCCAACTCATTTGCTCAC	CTCTAACCTCTGGGTCTGATCTT
Rim1	GGCACTCCAGAAAGTCTGAAAGAT	TCCTTGTCGGTGCGTTCTGT
Basson	ATGGATTTCCAGCCCACCAG	CAGGAGGGTAGGTAGGTGCT

### Histological analysis

The mice were sacrificed by cervical dislocation, and the eyes and cochleae were promptly and rapidly removed and fixed in paraformaldehyde fixative for 2 h at room temperature (RT), followed by overnight fixation in 10% neutral buffered formalin at 4° C. The eyes and cochleae were dehydrated by a series of ethanol washes ranging from 30–100% and embedded in paraffin. The paraffin-embedded tissues were sectioned at 7-um thickness, stained with hematoxylin and eosin (H&E), then viewed under light microscopy (Nikon YS100, Japan).

### Retina preparation and immunostaining analysis

Eyes were removed from wild type and *Piccolo*-/- mice and fixed in 4% paraformaldehyde overnight at 4° C. The fixed eyeballs were immersed in 15% sucrose for 4 h at RT then in 30% sucrose overnight at 4° C. Finally, the samples were frozen in optimal cutting temperature compound at -20° C. The tissues were sectioned at 7 μm thickness, washed with 10 mM PBS and blocked in 10% goat serum for 30 minutes at RT. The samples were incubated with primary antibodies overnight at 4° C and secondary antibody for 1 h at RT. Nuclear staining (DAPI) was applied to the samples for 10 minutes at RT. The labeled sections were imaged using a LSM 700 confocal microscope.

### Electroretinogram (ERG) detection in mice

The electroretinogram (ERG) is a complex electrical response, which is an indicator of retinal function. When the mice are stimulated with different intensities of light, a series of electrical signals, namely ERGs, can be detected on the oscilloscope. ERGs were measured and recorded using a Maxwellian-view Ganzfeld ERG system. Mice were dark-adapted for at least 12 hours, prior to the ERG experiment. Under dim red light, mice were anesthetized with 0.007 g/mL pentobarbital sodium by intraperitoneal injection (50 mg/kg body weight) as described earlier. Body temperature was maintained at approximately 37° C using a heating pad place on the ERG stage. The pupils were dilated with 1% tropicamide ophthalmic solution. The reference needle electrodes were gently inserted into each cheek and the ground electrode was placed just under the skin of the tail. Silver thread electrodes were placed on the corneal surface of each eye for recording the ERG responses. The test was performed under different flash intensities. The primary measurement was the amplitude of the signal. Statistical significance of comparisons between wild type and *Piccolo*-/- mice was calculated using a two-tailed Student’s t-Test. Mice used for ERGs were 1,4 and 8months of age.

### Transmission electron microscopy

Eyeballs were dissected and fixed in 2.5% glutaraldehyde for 30 minutes. The lens was then removed, and the rest of the optic cup was placed in a new fixative at 4° C overnight. The fixed optic cup was removed from the glutaraldehyde, washed three times with PBS, then post-fixed for 2 h in 1% osmium tetroxide. Samples were ethanol gradient dehydrated and embedded in Eps812 resin, and ultra-thin sections (70 nm) were cut on an ultramicrotome. The sections were stained with 2% uranyl acetate for 20 minutes and with lead citrate for 15 minutes, then observed under a JEOL-1200EX electron microscope at 80 Kv.

### Optical coherence tomography (OCT)

Optical coherence tomography (OCT) is an optical diagnostic technique that uses the interference of light to observe biological tissue structure. OCT has the advantages of nondestructive, rapid imaging with high resolution and can be used for *in vivo* examination of macula, nerve fiber layer, retina and optic disc. Mice were anesthetized with pentobarbital sodium, and their pupils were dilated with tropicamide and phenylephrine. Medical grade sodium hyaluronate was then applied to the cornea of the mouse eye, which was in contact with the Phoenix Micron IV retinal microscope lens. The eyes of the mice were photographed after adjusting the test bench and the focal length of the lens. After the experiment was completed, the eyes of the mice were washed with physiological saline, and levofloxacin eye drops were used to prevent infection.

### Statistical analysis

All data were from at least three independent experiments and are expressed as the mean ± SEM or SD to ensure the data accuracy and repeatability. Statistical analysis was performed using a two-tailed Student’s t-test. For all tests, P < 0.05 was considered statistically significant.

### Data availability statement

The data used to support the results of this study are available from the corresponding author upon request.

### Ethics statement

The experimental animals and operating procedures used in this paper are in accordance with the regulations of the Ethics Committee of Experimental Animals, College of Life Sciences, Shandong University (Permit Number: ECAESDUSM 20123004).

## Supplementary Material

Supplementary Figure 1

## References

[1] Moser T, Grabner CP, Schmitz F. Sensory processing at ribbon synapses in the retina and the cochlea. Physiol Rev. 2020; 100:103–44. 10.1152/physrev.00026.2018 31373863

[2] Zhai RG, Vardinon-Friedman H, Cases-Langhoff C, Becker B, Gundelfinger ED, Ziv NE, Garner CC. Assembling the presynaptic active zone: A characterization of an active one precursor vesicle. Neuron. 2001; 29:131–43. 10.1016/s0896-6273(01)00185-4 11182086

[3] Gundelfinger ED, Fejtova A. Molecular organization and plasticity of the cytomatrix at the active zone. Curr Opin Neurobiol. 2012; 22:423–30. 10.1016/j.conb.2011.10.005 22030346

[4] Zanazzi G, Matthews G. The molecular architecture of ribbon presynaptic terminals. Mol Neurobiol. 2009; 39:130–48. 10.1007/s12035-009-8058-z 19253034 PMC2701268

[5] Südhof TC. The presynaptic active zone. Neuron. 2012; 75:11–25. 10.1016/j.neuron.2012.06.012 22794257 PMC3743085

[6] Takao-Rikitsu E, Mochida S, Inoue E, Deguchi-Tawarada M, Inoue M, Ohtsuka T, Takai Y. Physical and functional interaction of the active zone proteins, CAST, RIM1, and Bassoon, in neurotransmitter release. J Cell Biol. 2004; 164:301–11. 10.1083/jcb.200307101 14734538 PMC2172332

[7] Waites CL, Leal-Ortiz SA, Andlauer TF, Sigrist SJ, Garner CC. Piccolo regulates the dynamic assembly of presynaptic F-actin. J Neurosci. 2011; 31:14250–63. 10.1523/JNEUROSCI.1835-11.2011 21976510 PMC3210199

[8] Fenster SD, Kessels MM, Qualmann B, Chung WJ, Nash J, Gundelfinger ED, Garner CC. Interactions between Piccolo and the actin/dynamin-binding protein Abp1 link vesicle endocytosis to presynaptic active zones. J Biol Chem. 2003; 278:20268–77. 10.1074/jbc.M210792200 12654920

[9] Waites CL, Leal-Ortiz SA, Okerlund N, Dalke H, Fejtova A, Altrock WD, Gundelfinger ED, Garner CC. Bassoon and piccolo maintain synapse integrity by regulating protein ubiquitination and degradation. EMBO J. 2013; 32:954–69. 10.1038/emboj.2013.27 23403927 PMC3616282

[10] Wagh D, Terry-Lorenzo R, Waites CL, Leal-Ortiz SA, Maas C, Reimer RJ, Garner CC. Piccolo directs activity dependent F-actin assembly from presynaptic active zones via Daam1. PLoS One. 2015; 10:e0120093. 10.1371/journal.pone.0120093 25897839 PMC4405365

[11] Fenster SD, Garner CC. Gene structure and genetic localization of the PCLO gene encoding the presynaptic active zone protein Piccolo. Int J Dev Neurosci. 2002; 20:161–71. 10.1016/s0736-5748(02)00046-1 12175852

[12] Regus-Leidig H, Ott C, Löhner M, Atorf J, Fuchs M, Sedmak T, Kremers J, Fejtová A, Gundelfinger ED, Brandstätter JH. Identification and immunocytochemical characterization of Piccolino, a novel Piccolo splice variant selectively expressed at sensory ribbon synapses of the eye and ear. PLoS One. 2013; 8:e70373. 10.1371/journal.pone.0070373 23936420 PMC3735604

[13] Regus-Leidig H, Fuchs M, Löhner M, Leist SR, Leal-Ortiz S, Chiodo VA, Hauswirth WW, Garner CC, Brandstätter JH. *In vivo* knockdown of Piccolino disrupts presynaptic ribbon morphology in mouse photoreceptor synapses. Front Cell Neurosci. 2014; 8:259. 10.3389/fncel.2014.00259 25232303 PMC4153300

[14] Matthews G, Fuchs P. The diverse roles of ribbon synapses in sensory neurotransmission. Nat Rev Neurosci. 2010; 11:812–22. 10.1038/nrn2924 21045860 PMC3065184

[15] tom Dieck S, Brandstätter JH. Ribbon synapses of the retina. Cell Tissue Res. 2006; 326:339–46. 10.1007/s00441-006-0234-0 16775698

[16] Francis HW, Rivas A, Lehar M, Ryugo DK. Two types of afferent terminals innervate cochlear inner hair cells in C57BL/6J mice. Brain Res. 2004; 1016:182–94. 10.1016/j.brainres.2004.05.016 15246854

[17] Martinez-Dunst C, Michaels RL, Fuchs PA. Release sites and calcium channels in hair cells of the chick’s cochlea. J Neurosci. 1997; 17:9133–44. 10.1523/JNEUROSCI.17-23-09133.1997 9364060 PMC6573622

[18] Meyer AC, Frank T, Khimich D, Hoch G, Riedel D, Chapochnikov NM, Yarin YM, Harke B, Hell SW, Egner A, Moser T. Tuning of synapse number, structure and function in the cochlea. Nat Neurosci. 2009; 12:444–53. 10.1038/nn.2293 19270686

[19] Kantardzhieva A, Liberman MC, Sewell WF. Quantitative analysis of ribbons, vesicles, and cisterns at the cat inner hair cell synapse: correlations with spontaneous rate. J Comp Neurol. 2013; 521:3260–71. 10.1002/cne.23345 23787810 PMC4309284

[20] Liberman LD, Wang H, Liberman MC. Opposing gradients of ribbon size and AMPA receptor expression underlie sensitivity differences among cochlear-nerve/hair-cell synapses. J Neurosci. 2011; 31:801–08. 10.1523/JNEUROSCI.3389-10.2011 21248103 PMC3290333

[21] Merchan-Perez A, Liberman MC. Ultrastructural differences among afferent synapses on cochlear hair cells: correlations with spontaneous discharge rate. J Comp Neurol. 1996; 371:208–21. 10.1002/(SICI)1096-9861(19960722)371:2<208::AID-CNE2>3.0.CO;2-6 8835727

[22] Ohn TL, Rutherford MA, Jing Z, Jung S, Duque-Afonso CJ, Hoch G, Picher MM, Scharinger A, Strenzke N, Moser T. Hair cells use active zones with different voltage dependence of Ca2+ influx to decompose sounds into complementary neural codes. Proc Natl Acad Sci USA. 2016; 113:E4716–25. 10.1073/pnas.1605737113 27462107 PMC4987782

[23] Fettiplace R. Hair cell transduction, tuning, and synaptic transmission in the mammalian cochlea. Compr Physiol. 2017; 7:1197–227. 10.1002/cphy.c160049 28915323 PMC5658794

[24] Heidelberger R, Thoreson WB, Witkovsky P. Synaptic transmission at retinal ribbon synapses. Prog Retin Eye Res. 2005; 24:682–720. 10.1016/j.preteyeres.2005.04.002 16027025 PMC1383430

[25] Graw J. Eye development. Curr Top Dev Biol. 2010; 90:343–86. 10.1016/S0070-2153(10)90010-0 20691855

[26] Amini R, Rocha-Martins M, Norden C. Neuronal migration and lamination in the vertebrate retina. Front Neurosci. 2018; 11:742. 10.3389/fnins.2017.00742 29375289 PMC5767219

[27] Hoon M, Okawa H, Della Santina L, Wong RO. Functional architecture of the retina: development and disease. Prog Retin Eye Res. 2014; 42:44–84. 10.1016/j.preteyeres.2014.06.003 24984227 PMC4134977

[28] Wert KJ, Skeie JM, Bassuk AG, Olivier AK, Tsang SH, Mahajan VB. Functional validation of a human CAPN5 exome variant by lentiviral transduction into mouse retina. Hum Mol Genet. 2014; 23:2665–77. 10.1093/hmg/ddt661 24381307 PMC3990166

[29] Hartong DT, Berson EL, Dryja TP. Retinitis pigmentosa. Lancet. 2006; 368:1795–809. 10.1016/S0140-6736(06)69740-7 17113430

[30] Daiger SP, Sullivan LS, Bowne SJ. Genes and mutations causing retinitis pigmentosa. Clin Genet. 2013; 84:132–41. 10.1111/cge.12203 23701314 PMC3856531

[31] Berson EL, Rosner B, Sandberg MA, Dryja TP. Ocular findings in patients with autosomal dominant retinitis pigmentosa and a rhodopsin gene defect (Pro-23-His). Arch Ophthalmol. 1991; 109:92–101. 10.1001/archopht.1991.01080010094039 1987956

[32] Wert KJ, Lin JH, Tsang SH. General pathophysiology in retinal degeneration. Dev Ophthalmol. 2014; 53:33–43. 10.1159/000357294 24732759 PMC4405532

[33] Chang S, Vaccarella L, Olatunji S, Cebulla C, Christoforidis J. Diagnostic challenges in retinitis pigmentosa: genotypic multiplicity and phenotypic variability. Curr Genomics. 2011; 12:267–75. 10.2174/138920211795860116 22131872 PMC3131734

[34] Mukherjee K, Yang X, Gerber SH, Kwon HB, Ho A, Castillo PE, Liu X, Südhof TC. Piccolo and bassoon maintain synaptic vesicle clustering without directly participating in vesicle exocytosis. Proc Natl Acad Sci USA. 2010; 107:6504–09. 10.1073/pnas.1002307107 20332206 PMC2851964

[35] Jiang L, Wei Y, Ronquillo CC, Marc RE, Yoder BK, Frederick JM, Baehr W. Heterotrimeric kinesin-2 (KIF3) mediates transition zone and axoneme formation of mouse photoreceptors. J Biol Chem. 2015; 290:12765–78. 10.1074/jbc.M115.638437 25825494 PMC4432293

[36] Jimeno D, Feiner L, Lillo C, Teofilo K, Goldstein LS, Pierce EA, Williams DS. Analysis of kinesin-2 function in photoreceptor cells using synchronous Cre-loxP knockout of Kif3a with RHO-Cre. Invest Ophthalmol Vis Sci. 2006; 47:5039–46. 10.1167/iovs.06-0032 17065525 PMC1904505

[37] Turner J, Crossley M. The CtBP family: enigmatic and enzymatic transcriptional co-repressors. Bioessays. 2001; 23:683–90. 10.1002/bies.1097 11494316

[38] Katsanis N, Fisher EM. A novel C-terminal binding protein (CTBP2) is closely related to CTBP1, an adenovirus E1A-binding protein, and maps to human chromosome 21q21.3. Genomics. 1998; 47:294–99. 10.1006/geno.1997.5115 9479502

[39] Johnson S, Halford S, Morris AG, Patel RJ, Wilkie SE, Hardcastle AJ, Moore AT, Zhang K, Hunt DM. Genomic organisation and alternative splicing of human RIM1, a gene implicated in autosomal dominant cone-rod dystrophy (CORD7). Genomics. 2003; 81:304–14. 10.1016/s0888-7543(03)00010-7 12659814

[40] Michaelides M, Holder GE, Hunt DM, Fitzke FW, Bird AC, Moore AT. A detailed study of the phenotype of an autosomal dominant cone-rod dystrophy (CORD7) associated with mutation in the gene for RIM1. Br J Ophthalmol. 2005; 89:198–206. 10.1136/bjo.2004.050773 15665353 PMC1772528

[41] Dick O, tom Dieck S, Altrock WD, Ammermüller J, Weiler R, Garner CC, Gundelfinger ED, Brandstätter JH. The presynaptic active zone protein bassoon is essential for photoreceptor ribbon synapse formation in the retina. Neuron. 2003; 37:775–86. 10.1016/s0896-6273(03)00086-2 12628168

[42] Falck J, Bruns C, Hoffmann-Conaway S, Straub I, Plautz EJ, Orlando M, Munawar H, Rivalan M, Winter Y, Izsvák Z, Schmitz D, Hamra FK, Hallermann S, et al. Loss of Piccolo function in rats induces cerebellar network dysfunction and pontocerebellar hypoplasia type 3-like phenotypes. J Neurosci. 2020; 40:2943–59. 10.1523/JNEUROSCI.2316-19.2020 32122952 PMC7117892

[43] Ahmed MY, Chioza BA, Rajab A, Schmitz-Abe K, Al-Khayat A, Al-Turki S, Baple EL, Patton MA, Al-Memar AY, Hurles ME, Partlow JN, Hill RS, Evrony GD, et al. Loss of PCLO function underlies pontocerebellar hypoplasia type III. Neurology. 2015; 84:1745–50. 10.1212/WNL.0000000000001523 25832664 PMC4424132

[44] Juranek JK, Mukherjee K, Siddiqui TJ, Kaplan BJ, Li JY, Ahnert-Hilger G, Jahn R, Calka J. Active zone protein expression changes at the key stages of cerebellar cortex neurogenesis in the rat. Acta Histochem. 2013; 115:616–25. 10.1016/j.acthis.2013.01.003 23434052

[45] Müller TM, Gierke K, Joachimsthaler A, Sticht H, Izsvák Z, Hamra FK, Fejtová A, Ackermann F, Garner CC, Kremers J, Brandstätter JH, Regus-Leidig H. A multiple Piccolino-RIBEYE interaction supports plate-shaped synaptic ribbons in retinal neurons. J Neurosci. 2019; 39:2606–19. 10.1523/JNEUROSCI.2038-18.2019 30696732 PMC6445989

[46] Dagostin A, Kushmerick C, von Gersdorff H. Allegro giusto: piccolo, bassoon and clarinet set the tempo of vesicle pool replenishment. J Physiol. 2018; 596:1315–16. 10.1113/JP275704 29446080 PMC5899974

[47] Limbach C, Laue MM, Wang X, Hu B, Thiede N, Hultqvist G, Kilimann MW. Molecular *in situ* topology of Aczonin/Piccolo and associated proteins at the mammalian neurotransmitter release site. Proc Natl Acad Sci USA. 2011; 108:E392–401. 10.1073/pnas.1101707108 21712437 PMC3150911

[48] Moser T, Brandt A, Lysakowski A. Hair cell ribbon synapses. Cell Tissue Res. 2006; 326:347–59. 10.1007/s00441-006-0276-3 16944206 PMC4142044

[49] Regus-Leidig H, Brandstätter JH. Structure and function of a complex sensory synapse. Acta Physiol (Oxf). 2012; 204:479–86. 10.1111/j.1748-1716.2011.02355.x 21880116

[50] Regus-Leidig H, tom Dieck S, Brandstätter JH. Absence of functional active zone protein Bassoon affects assembly and transport of ribbon precursors during early steps of photoreceptor synaptogenesis. Eur J Cell Biol. 2010; 89:468–75. 10.1016/j.ejcb.2009.12.006 20188438

[51] Gundelfinger ED, Reissner C, Garner CC. Role of Bassoon and piccolo in assembly and molecular organization of the active zone. Front Synaptic Neurosci. 2016; 7:19. 10.3389/fnsyn.2015.00019 26793095 PMC4709825

[52] Ivanova D, Dirks A, Fejtova A. Bassoon and piccolo regulate ubiquitination and link presynaptic molecular dynamics with activity-regulated gene expression. J Physiol. 2016; 594:5441–48. 10.1113/JP271826 26915533 PMC5043050

[53] Fenster SD, Chung WJ, Zhai R, Cases-Langhoff C, Voss B, Garner AM, Kaempf U, Kindler S, Gundelfinger ED, Garner CC. Piccolo, a presynaptic zinc finger protein structurally related to bassoon. Neuron. 2000; 25:203–14. 10.1016/s0896-6273(00)80883-1 10707984

[54] Dick O, Hack I, Altrock WD, Garner CC, Gundelfinger ED, Brandstätter JH. Localization of the presynaptic cytomatrix protein Piccolo at ribbon and conventional synapses in the rat retina: comparison with Bassoon. J Comp Neurol. 2001; 439:224–34. 10.1002/cne.1344 11596050

[55] Brandstätter JH, Fletcher EL, Garner CC, Gundelfinger ED, Wässle H. Differential expression of the presynaptic cytomatrix protein bassoon among ribbon synapses in the mammalian retina. Eur J Neurosci. 1999; 11:3683–93. 10.1046/j.1460-9568.1999.00793.x 10564375

[56] Hamel C. Retinitis pigmentosa. Orphanet J Rare Dis. 2006; 1:40. 10.1186/1750-1172-1-40 17032466 PMC1621055

[57] Raghupathy RK, Zhang X, Alhasani RH, Zhou X, Mullin M, Reilly J, Li W, Liu M, Shu X. Abnormal photoreceptor outer segment development and early retinal degeneration in kif3a mutant zebrafish. Cell Biochem Funct. 2016; 34:429–40. 10.1002/cbf.3205 27470972

[58] Fu X, Zhang L, Jin Y, Sun X, Zhang A, Wen Z, Zhou Y, Xia M, Gao J. Loss of Myh14 increases susceptibility to noise-induced hearing loss in CBA/CaJ mice. Neural Plast. 2016; 2016:6720420. 10.1155/2016/6720420 28101381 PMC5215640

[59] Li P, Wen Z, Zhang G, Zhang A, Fu X, Gao J. Knock-in mice with Myo3a Y137C mutation displayed progressive hearing loss and hair cell degeneration in the inner ear. Neural Plast. 2018; 2018:4372913. 10.1155/2018/4372913 30123247 PMC6079384

[60] Fu X, Sun X, Zhang L, Jin Y, Chai R, Yang L, Zhang A, Liu X, Bai X, Li J, Wang H, Gao J. Tuberous sclerosis complex-mediated mTORC1 overactivation promotes age-related hearing loss. J Clin Invest. 2018; 128:4938–55. 10.1172/JCI98058 30247156 PMC6205401

