# Supplementary material for: Piccolo is essential for the maintenance of mouse retina but not cochlear hair cell function
Source: Aging (Albany NY). 2021 Apr 21;13(8):11678–95. doi: 10.18632/aging.202861 (PMC8109093; doi:10.18632/aging.202861)
Supplement: Supplementary Figure 1 [file aging-13-202861-s001.pdf]

SUPPLEMENTARY FIGURE

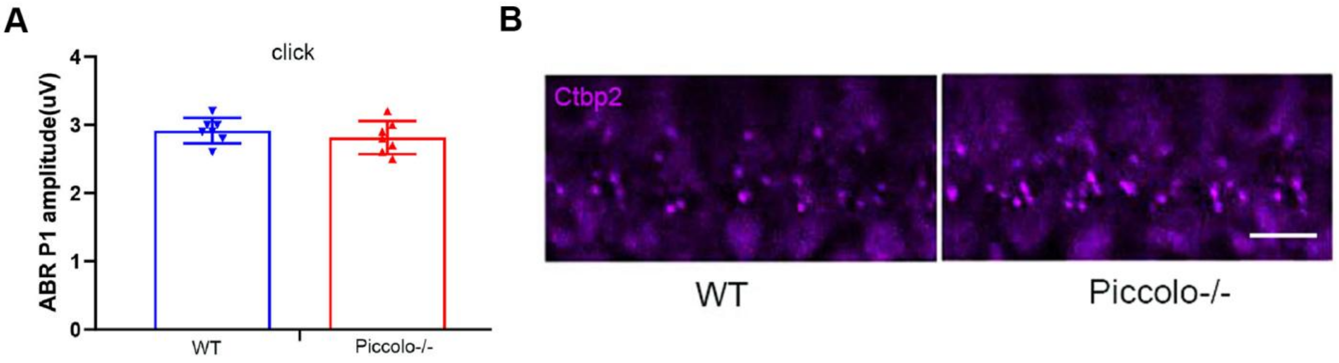

Supplementary Figure 1. (**A**) The peak I amplitudes of the ABR waves show no significant difference in both *Piccolo*-/- and wild-type mice. (**B**) The immunofluorescence result showed normal ribbon synapses in the inner hair cells of *Piccolo*-/- mice at P120. The ribbon synapse were immustained by Ctbp2. Scale bar: 5um.
